# IR Sensors, Related Materials, and Applications

**DOI:** 10.3390/s25030673

**Published:** 2025-01-23

**Authors:** Nikolaos Argirusis, Achilleas Achilleos, Niyaz Alizadeh, Christos Argirusis, Georgia Sourkouni

**Affiliations:** 1 MAT4NRG GmbH, 38678 Clausthal-Zellerfeld, Germany; niyaz.alizadeh@mat4nrg.de; 2 Department of Mechanical Engineering, Division LMSD, KU Leuven, Celestijnenlaan 300, Box 2420, 3001 Leuven, Belgium; achilleas.achilleos@kuleuven.be; 3 School of Chemical Engineering, National Technical University of Athens (NTUA), 15773 Zografou-Athens, Greece; 4 Clausthal Centre of Materials Technology, TU Clausthal, 38678 Clausthal-Zellerfeld, Germany; cogsa@tu-clausthal.de

**Keywords:** infrared sensors, infrared sensing materials, infrared sensor types, sensor applications, thermal detectors, photoelectric detectors

## Abstract

Infrared (IR) sensors are widely used in various applications due to their ability to detect infrared radiation. Currently, infrared detector technology is in its third generation and faces enormous challenges. IR radiation propagation is categorized into distinct transmission windows with the most intriguing aspects of thermal imaging being mid-wave infrared (MWIR) and long-wave infrared (LWIR). Infrared detectors for thermal imaging have many uses in industrial applications, security, search and rescue, surveillance, medical, research, meteorology, climatology, and astronomy. Presently, high-performance infrared imaging technology mostly relies on epitaxially grown structures of the small-bandgap bulk alloy mercury–cadmium–telluride (MCT), indium antimonide (InSb), and GaAs-based quantum well infrared photodetectors (QWIPs), contingent upon the application and wavelength range. Nanostructures and nanomaterials exhibiting appropriate electrical and mechanical properties including two-dimensional materials, graphene, quantum dots (QDs), quantum dot in well (DWELL), and colloidal quantum dot (CQD) will significantly enhance the electronic characteristics of infrared photodetectors, transition metal dichalcogenides, and metal oxides, which are garnering heightened interest. The present manuscript gives an overview of IR sensors, their types, materials commonly used in them, and examples of related applications. Finally, a summary of the manuscript and an outlook on prospects are given.

## 1. Introduction

Infrared (IR) radiation was discovered approximately 200 years ago but infrared photodetectors (IRPDs) were developed in the late 20th century. Infrared photodetectors (IRPDs) have emerged as crucial technologies in diverse applications, including night vision, military missile tracking, medical imaging, industrial flaw detection, environmental monitoring, and planetary discovery. A primary challenge in photonics is the advancement of optoelectronic technology and optical signal detection systems. A crucial component of photonics is the photosensor. The sensor’s architecture comprises a photosensitive element that converts optical radiation into an electrical signal, together with electronic gain and pre-processing circuits for signal transmission, conversion, and information storage in either analog or digital format. The primary metrological attributes of photosensors include sensitivity, accuracy, and stability with time, which refers to the capacity to sustain a specified accuracy and reproducibility of measurement outcomes over an extended duration (calibration interval) [1,2].

The infrared spectrum is categorized into short-wave infrared (SWIR, 1–3 µm), mid-wave infrared (MWIR, 3–5 µm), long-wave infrared (LWIR, 8–12 µm), and very long-wave infrared (VLWIR, >12 µm) spectral bands. Infrared detectors can be categorized as either photon detectors, thermal detectors, or radiation field detectors. In the initial category, photons directly interact with the charge carriers in a semiconductor to produce a photocurrent. The second group is defined by alterations in certain material properties resulting from temperature changes induced by the absorption of infrared radiation [3].

**Figure 1:**
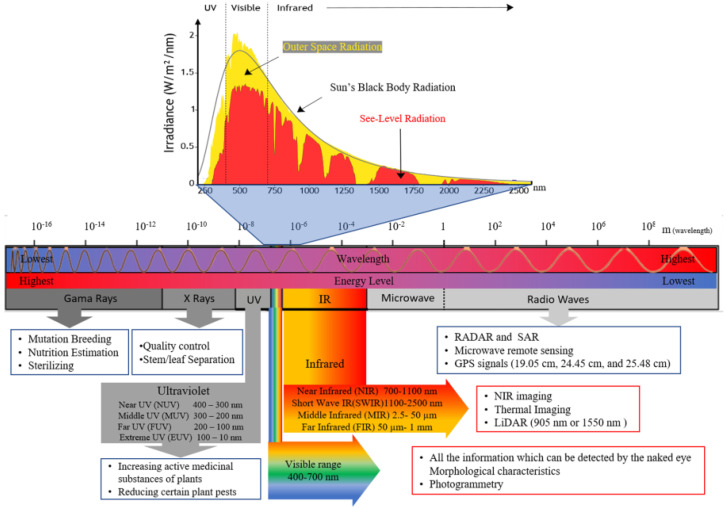
The electromagnetic spectrum with indicative regions and energy levels as well as indicative applications (adapted from [4] with permission).

Every section of the electromagnetic spectrum conveys valuable information for a particular purpose (Figure 1). The visible range of 0.4–0.7 µm is utilized for vision-based sensing applications, including chlorophyll research, green indices, morphological analysis of leaves and fruits, and vision-based metrics to quantify all data discernible by the naked eye. Conversely, the infrared spectrum is utilized to obtain data that are otherwise imperceptible, including assessments of plant water content and stress-related research. Figure 1 illustrates the electromagnetic spectrum, delineating distinct zones along with corresponding agricultural applications for each segment. The boundaries delineating electromagnetic areas are not distinctly defined and they can fluctuate according to the application [4].

In the present manuscript, besides an overview on traditional IR materials and sensors, an evaluation of the current situation and forecasts about future advancements of quantum dot infrared photodetectors (QDIPs) are given. Further a comparison of traditional materials for IR sensors and modern materials like HgCdTe photodiodes, quantum well infrared photodetectors (QWIPs), type II superlattice photodiodes, Schottky barrier photoemissive detectors, doped silicon detectors, and high-temperature superconductor detectors will be presented.

In the present manuscript, we present types of IR sensors and related materials. We discuss the development till today, the future prospects, and give examples on present and future applications.

## 2. Types of IR Sensors

There are three categories of devices that detect infrared irradiation: photon, thermal, and radiation field detectors. Further, infrared sensors can be largely divided into “thermal” and “quantum” types based on their operational principles. Thermal sensors convert infrared rays into heat which is then changed to a resistance change and thermoelectromotive force to extract the output. Quantum sensors use the photoconductive effect based on the transitional energy of semiconductors and the photovoltaic effect in PN junctions. Pyroelectric infrared sensors fall under the thermal type of sensors.

### 2.1. Active and Passive Infrared Sensors

Every object emits electromagnetic energy when their absolute temperature exceeds absolute zero, referred to as blackbody radiation. The sun, with an absolute temperature of about 5800 K, serves as the principal source of energy and electromagnetic radiation on Earth. Solar radiation predominantly consists of visible light and infrared radiation, with a peak wavelength approximately at 500 nm. Furthermore, the Earth, possessing a temperature of 290 K, emits feeble radiation across a broad spectrum, peaking at approximately 10 µm. Consequently, passive sensors rely on either solar or terrestrial radiation and are engineered to operate in spectral regions with the highest natural radiation energy. Thermal detectors are typically engineered to be responsive to radiation between 7 µm and 14 µm [5].

**Figure 2:**
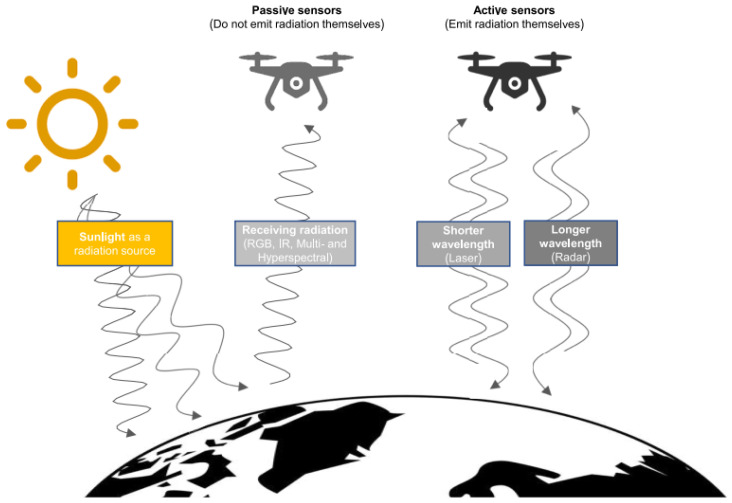
Schematic of unmanned aerial vehicles with passive and active sensors. (Reprinted from [6] with permission).

Active infrared sensors (AIR) transmit and receive (Figure 2) in the infrared part of the spectrum. It comprises two components: a light-emitting source and a receiver. As an object approaches the sensor, the infrared light emitted by the IR-source reflects off the object and is captured by the receiver. Active infrared sensors function as proximity sensors and are frequently employed in obstacle detection systems, such as those in robotics.

Passive IR sensors exclusively detect infrared radiation and do not emit it via a source. Passive infrared sensors (PIR) consist of the following components: two layers of pyroelectric material (a pyroelectric sensor); an IR filter to exclude all other wavelengths of light; a Fresnel lens that converges light from multiple angles into a single point; and a housing unit that safeguards the sensor from environmental variables, such as temperature and humidity. PIR sensors are primarily utilized in PIR-based motion detection systems.

### 2.2. Extrinsic and Intrinsic Infrared Sensors

The performance of photoconductive, photovoltaic, and photoelectromagnetic detectors is fundamentally reliant on the lifespan of photoexcited carriers.

**Figure 3:**
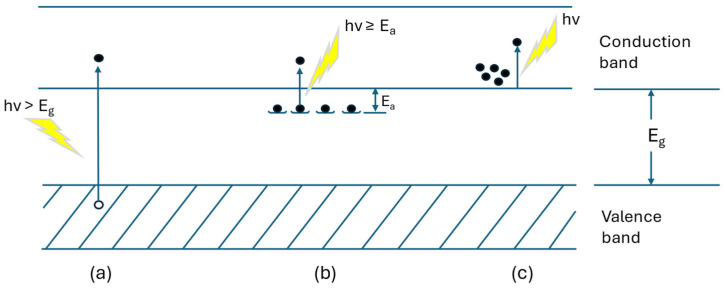
Fundamental optical excitation processes in semiconductors: (**a**) intrinsic absorption, (**b**) extrinsic absorption, and (**c**) free carrier absorption (after [7,9] with permission).

In photon detectors, incident photons are absorbed by interacting with electrons, which may be related to lattice atoms, impurity atoms, or even free electrons. The measured signal arises from the altered distribution of electronic energy. Figure 3 illustrates the fundamental optical excitation mechanisms in semiconductors [7]. In this respect, it is important to clarify that the terms extrinsic and intrinsic are meant as mentioned in [6] and not as intrinsic and/or extrinsic components in complete devices by Kwon et al. [8].

Remote sensing is the discipline of obtaining and analyzing data on objects without direct interaction. It encompasses the detection and documentation of reflected or emitted energy across various wavelengths, followed by the processing and analysis of the data to transform them into knowledge.

IR sensors are generally classified into two categories: photon detectors, which typically require cooling, and thermal detectors, which may not necessitate cooling.

Uncooled infrared (IR) sensors for the detection of mid-to-long wavelength IR light typically rely on thermal detection principles. In thermal detectors, infrared radiation is absorbed, transformed into heat, and measured by a thermometer. Determining the thermometer’s temperature allows for the estimation of the flux of incoming infrared radiation [10]. Mid-infrared fiber optic accessories have demonstrated applicability in a diverse range of chemical studies, especially in the pharmaceutical sector.

Photon detectors typically consist of semiconductor devices featuring photoconductors, Schottky diodes, quantum well IR photodetectors, or photovoltaic devices as sensing elements. In contrast, thermal detectors utilize sensing elements such as thermocouples, bolometers, Golay cells, and pyroelectric devices [11].

## 3. Materials for IR Sensors

### 3.1. Organic and Carbon-Based Materials

Organic materials have emerged as prominent subjects of study in pyroelectric materials, and with the progress in polymer-based substances, numerous functional polymer materials have demonstrated ferroelectric, piezoelectric, and pyroelectric capabilities as well [12,13]. Polyvinylidene fluoride (PVDF) is one of the most thoroughly researched pyroelectric materials, exhibiting superior pyroelectric and piezoelectric coefficients of 25 µC m^−2^ K^−1^ and 33 ρCN^−1^, respectively, in comparison to other polymeric substances. The low dielectric constant (εr = 9) and elevated temperature stability (about 120 °C) yield an enhanced figure of merit.

Several studies have deployed organic-inorganic composites like PVDF and P(VDF)-TrFE as a matrix with insulating lead zirconate titanate (PZT) in piezoelectric and pyroelectric devices [14]. In Table 1 (from [12]), properties of different organic and organic–inorganic composite materials are tabulated.

**Figure 4:**
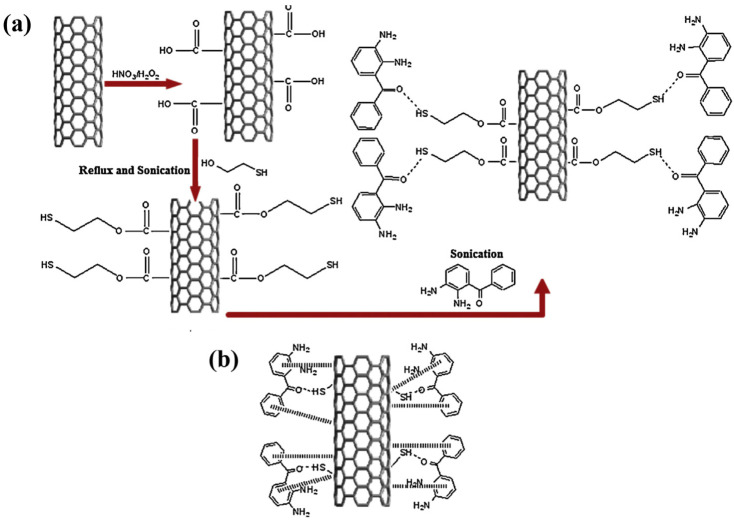
(**a**) Reaction scheme for functionalization and cross-linking of MWCNTs; (**b**) π-π stacking scheme. (Reproduced from [32] with permission).

Afrin et al. [32] have effectively developed an infrared sensor device utilizing functionalized multiwall carbon nanotube buckypapers by adjusting the ratio of the cross-linker reagent in the buckypapers. The carbon nanotubes were functionalized with carboxyl by sonication in concentrated hydrochloric acid and thiol groups by 3 h of sonication of previously carboxylated CNTs with 2-mercatoethanol (Figure 4). FT-IR and Raman spectroscopy analyses validated the attachment of the functional groups. SEM investigation revealed the shape and aggregation of nanotubes within the buckypapers. TEM investigation revealed the external wall imperfections induced by chemical processing of the carbon nanotubes. The MWCNT–COOH exhibits lower resistance (0.5 kΩ) compared to carboxylic and thiolated nanotube buckypapers (CNTTBPs). CNTTBPs exhibit a high resistance of 3.1 kΩ, resulting in less current flow compared to CNTCBPs, which possess a resistance of 1.6 kΩ. The increase in resistance in buckypapers was attributed to a higher ratio of cross-linker reagents in the nanotubes. The augmentation in resistance of the sensing material was confirmed using infrared sensing of buckypapers. The infrared sensor of thiolated buckypaper exhibited superior sensitivity compared to carboxylated bucky paper.

**Figure 5:**
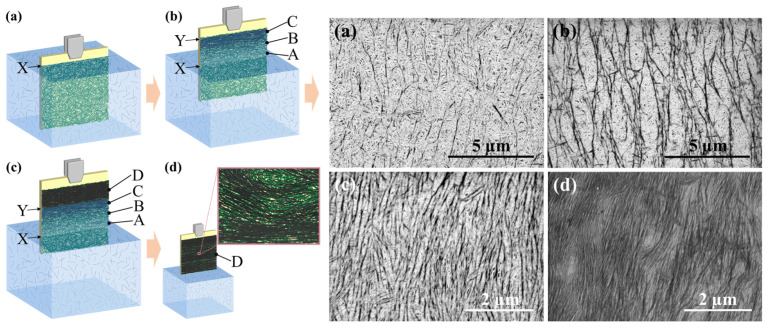
(**Left**) Images depicting the production process of a two-layer film and SEM images illustrating the adhesion process of the SWCNT on the wetted surface. (**a**) The lower layer is established within the dispersion. (**b**) The upper layer is progressively established in the wetted region approximately 5 mm above the dispersion interface. (**c**) Continuous desiccation of the wetted area. (**d**) Completely desiccated state resulting in the creation of a bilayer coating. (**Right**) (**a**) SEM picture of state A, where the upper layer has commenced adhesion. (**b**) SEM picture of condition B, whereby SWCNTs progressively adhere to occupy the interstitial spaces between aligned SWCNTs. (**c**) SEM picture of C exhibiting increased density in orientation. (**d**) SEM image of D in its fully desiccated condition. (Reproduced from [33] with permission).

Kosaka and coworkers [33] have realized uncooled IR sensors by manufacturing a bolometer based on two single-walled carbon nanotubes layers. The dip-coating technique has effectively generated a distinctive film comprising two layers with varying morphologies: an upper layer of aligned SWCNT film and a lower layer of a film of non-aligned SWCNT. In the top layer, single-walled carbon nanotubes (SWCNTs) aggregate into bundles of varying thicknesses, which subsequently create thin, aligned films of SWCNTs measuring 2–7 nm in thickness, characterized by parallel and uniformly spaced bundles (Figure 5). Reduced resistance was attained owing to the bundling and orientation of SWCNTs, along with the extensive connection area with the electrodes, while a TCR above −5%/K was exhibited in the fabricated device. As illustrated in Figure 5b (left), when the substrate was extracted from the dispersion interface X, the dispersion retained moisture on the substrate surface from the liquid interface X to Y. In this thin liquid phase on the substrate (between the interfaces X and Y), SWCNTs oriented at the liquid interface X maintained their orientation and progressively adhered to the lower-layer SWCNTs to occupy the gap (Figure 5b (left) A–C). The substrate was subsequently elevated, and by the time of the complete drying of the substrate at Y, the density of the aligned SWCNTs increased, resulting in the formation of an upper layer (Figure 5c (left) D). The ongoing development of the upper layer from A to D yielded the consistent development of a dense, aligned superior layer (Figure 5d (left)). In Figure 5 (right), SEM pictures of the aligned CNTs are shown.

### 3.2. Inorganic Materials

Non-crystalline films of (Ge_2_S_8_)_100−x_(As_2_Te_3_)_x_ (GSAT) (0 ≤ x ≤ 100) have been synthesized via the usual melting quench process by Alsaif et al. [34]. GSAT films were thermally placed onto chemically cleaned glass substrates. GSAT glasses are advantageous candidates for the application of optoelectronics, infrared fibers, and energy storage. The As_2_Te_3_ material possesses a narrow optical bandgap (Eg = 0.92 eV), leading to increased optical conductivity and mobility. Consequently, it holds potential for many optical devices, including optical sensors, switches, lenses, mirrors, infrared sensors, and detectors.

Lead zirconate titanate (PZT) and ferroelectrica

Recently, thermal-type pyroelectric infrared sensors utilizing PZT films have garnered significant interest in military, homeland security, and civilian sectors due to their cost-effectiveness, compactness, broad response spectrum, and superior reliability relative to conventional photon infrared detectors. Research on infrared devices utilizing PZT films encompasses night vision imagers, gas sensors, motion detectors, and more [35].

Ferroelectric lead zirconate titanate (PZT) 30/70 has exceptional dielectric characteristics and is suitable for use as PZT thin films in uncooled pyroelectric infrared sensors. A variety of uncooled pyroelectric sensors with diverse architectures have been engineered and developed to enhance their performance. Advanced MEMS technology has been employed to examine bulk and surface micro-machined micro-IR sensors [36,37] in order to minimize the thermal mass beneath the sensing element and to combine the IR-sensing thin film with its control circuits on a silicon chip as a cohesive device. Nickel has been effectively utilized as an absorption and selective layer in ferroelectric thin film pyroelectric sensors. The absorption of the Ni/PZT/Pt multilayer sensor to a He-Ne laser and infrared light can be optimized by modifying the thickness of either the Ni or PZT thin films [38].

PZT films with 1 µm thickness, with varying Nb doping levels, were formed on Pt(111)/TiO_2_/SiO_2_/Si(100) substrates using chemical solution deposition (CSD) and subsequently subjected to rapid thermal annealing (RTA). The implementation of the initial seeding layer markedly enhanced the crystallization quality post-RTA. The dielectric constant of PNZT films augmented with the elevation of Nb doping concentration. PZT films with 1% Nb doping demonstrate enhanced remnant polarization (49.33 µC/cm^2^), pyro-electric coefficient (4.6 × 10^−4^ C/K m^2^), and figure of merit (1.89 × 10^−5^ J m^3^ K^−1^), suggesting that Pb(Zr_0.2_Ti_0.8_)_0.99_-Nb_0.01_O_3_ is a promising option for pyroelectric infrared sensor applications [39].

Ferroelectric capacitive sensors integrated with organic field effect transistors on flexible substrates are appropriate for many sensing applications [40]. The direct integration of an organic field effect transistor (OFET) or an electrochemical transistor (ECT) with a fluoropolymer sensor element like polyvinylidene fluoride (PVDF) yields a pyroelectric sensor response, illustrating that pyroelectric sensors constructed from PVDF family polymers consist of a piezo- and/or pyroelectric polymer thin film capacitor integrated with high-performance organic thin film transistors (OTFTs) or ECTs that function as impedance converters, signal amplifiers, and conditioners as has been demonstrated by Zirkl and coworkers [41]. A wet-chemical production technique utilizing the sol–gel method has been devised to obtain ferroelectric thin films composed of PVDF and its copolymer trifluoroethylene (TrFE). The solutions exhibit favorable characteristics concerning various coating and printing procedures. The acquired layers exhibit favorable remnant polarizations and adequate characteristics concerning thermally induced voltage and current responses. Similar holds for the use of single-walled carbon nanotubes (SWCNTs) as reported by Park and coworkers [40], in the case of SWCNTs-based field effect transistors exhibiting a notable photoresponse, particularly a gate-enhanced photocurrent and an exciton–phonon coupled transition. This research will facilitate the development of SWCNT-based visual sensors with gate control capabilities.

Schottky photo-tunable barrier detector (SPBD)

**Figure 6:**
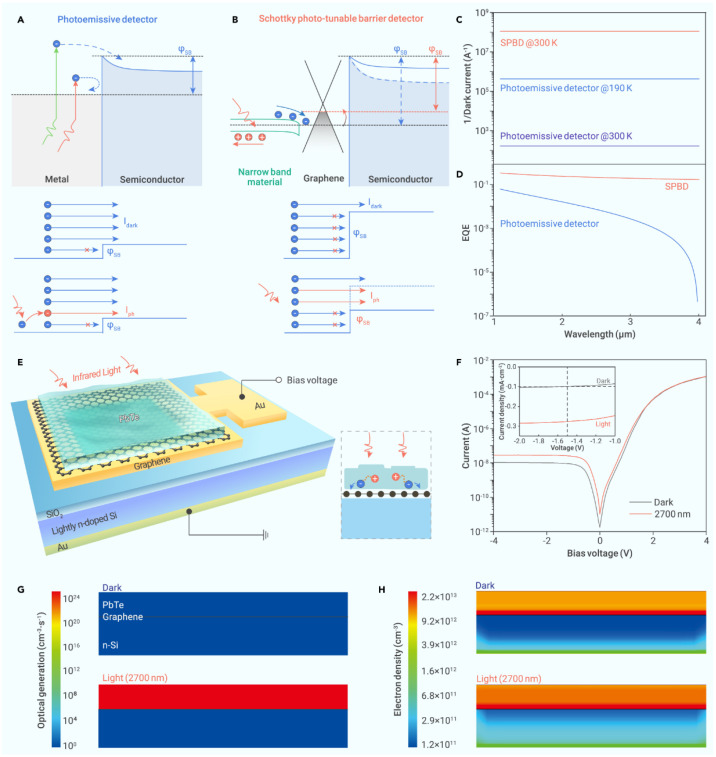
Conceptualization and attributes of the SPBD working mechanism of (**A**) a typical photoemissive detector featuring a metal–semiconductor junction and (**B**) the proposed SPBD presented herein. φSB, Idark, and Iph represent the Schottky barrier height, dark current, and photocurrent, respectively. The green (red) wavy arrow denotes light characterized by high (low) photon energy. (**C**) Dark current as a function of wavelength for the photoemissive detector and SPBD. The dark current, influenced by φSB, temperature, and bias voltage, remains consistent across various light wavelengths. (**D**) EQE versus wavelength plots for the photoemissive detector and SPBD. The spectral response of the narrow bandgap material in the analysis model is considered to be independent of wavelength. (**E**) Schematic representation of the SPBD. Infrared light induces the excitation of electron–hole pairs in PbTe. Photogenerated electrons will be injected into graphene, resulting in an increase in the Fermi level of the material. (**F**) I–V curves of the SPBD in both dark conditions and under 2700 nm illumination. Smooth I–V curves indicate effective contact between the upper Au layer and graphene as well as between the lower Au layer and Si. The rise in current under infrared illumination is due to the reduction in the Schottky barrier height (SBH). As the GrapheneSi junction is progressively forward-biased, a significant increase in forward current predominates, eclipsing the incremental current induced by infrared light. (**G**) Simulated optical generation and (**H**) electron density profile under 2700 nm illumination, compared to the equilibrium state in darkness. (Reproduced from [42] permission).

Fu and colleagues [42] suggested an uncooled Schottky photo-tunable barrier detector (SPBD) to surpass the internal photoemission limit, facilitating broadband and extremely sensitive photodetection capabilities. The SPBD arrangement comprises a graphene–silicon Schottky junction for carrier inhibition and a narrow bandgap lead telluride (PbTe) for IR light absorption. The photoresponse of the SPBD fundamentally differs from that of traditional Schottky infrared detectors (Figure 6), arising from the alteration in the Schottky barrier when exposed to infrared light. The operational mechanism of the SPBD in contrast to that of a conventional photoemission detector is illustrated in Figure 6A,B. The photoresponse of a photoemissive detector is primarily governed by internal photoemission. The analysis models of the photoemission detector and SPBD indicate that the SPBD can sustain a reduced dark current compared to a photoemissive detector functioning at ambient temperature or even under cryogenic settings by utilizing the substantial Schottky barrier height (Figure 6C). Moreover, as photon energy diminishes, the energy of the hot electrons declines, resulting in a reduced emission of hot electrons across the barrier in the photoemissive detector. Consequently, the external quantum efficiency (EQE) of the photoemissive detector diminishes as the light wavelength increases. The reduction in the SBH under illumination for the SPBD facilitates a significant influx of electrons across the barrier, leading to a comparatively elevated EQE in relation to the photoemissive detector (Figure 6D). Furthermore, a substantial Schottky barrier can be sustained to mitigate dark currents. Consequently, infrared light with photon energy beneath the threshold can be detected without compromising detectivity. The SPBD demonstrates broadband detection (254–4000 nm), a low dark current density (<10^−3^ A/cm^2^), and a rapid response time (0.13 ms/0.11 ms). Significantly, the device exhibits a room-temperature specific detectivity of up to 7.2 × 10^9^ cm Hz^−1/2^ W^−1^ under blackbody radiation, serving as a dependable method for assessing the performance of infrared photodetectors by simulating real-world objects [43].

Quantum dot infrared photodetectors (QDIPs)

Quantum dots (QDs) represent a class of structures that have garnered significant attention in recent years, owing to their diverse electronic and optoelectronic properties, which have been explored both theoretically and experimentally [44]. This creates multiple avenues for exploration, including military imaging and medical applications [45], as well as advancements in optoelectronics like infrared photodetectors, quantum dot lasers, quantum light emitting diodes, and fiber optic communications [46]. Quantum dots are nanoparticles measuring in the nanometer range, and by altering their size and shape, one can effectively fine-tune the bandgap energy of these nanostructures [47].

**Figure 7:**
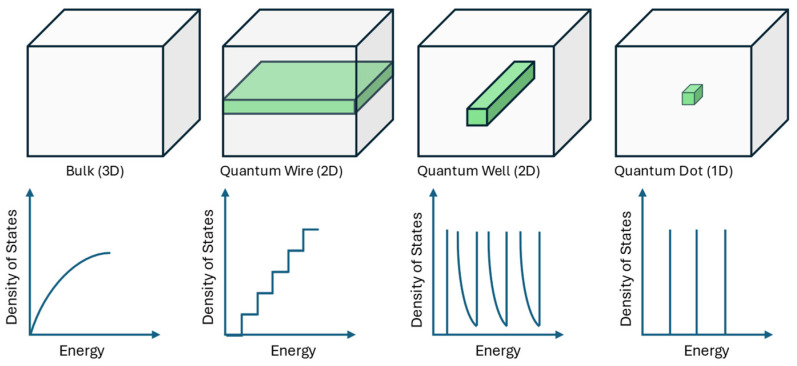
Schematic spatial distribution and density of states for bulk materials, quantum wells, quantum wires, and quantum dots.

A quantum dot is basically a self-contained three-dimensional island of semiconductor material, usually situated within another semiconductor matrix. Due to the different bandgaps of the two materials, the dots can “constrain” carriers (electrons, holes, or excitons) to a confined area, specifically the volume of the dot. Quantum dots have numerous parallels to quantum wells and quantum wires, both of which were recognized and studied before the emergence of quantum dots. A quantum wire is a slender column of one material embedded within a matrix of another. In this scenario, carriers are confined to one-dimensional movement along the wire’s length, owning solely that solitary degree of freedom (Figure 7).

Quantum dot systems exhibit zero-dimensional quantum confinement. In the absence of external influence, carriers will remain restricted within the confines of the dots in all three dimensions. The carriers will be restricted to a specified area within the semiconductor device. Furthermore, by modifying external variables, including the electric field and device temperature, carriers can be selectively captured or liberated.

This enables accurate control of the system’s attributes. The second property is that quantum dots exhibit atom-like properties. Their density of states, illustrated below, demonstrates a delta-like feature, setting it apart from bulk materials and other semiconductor structures like quantum wells and wires. The energy levels in quantum dots are distinctly defined and quantized, akin to the confinement of electrons in atoms, but with configurable attributes.

QDIPs are basically analogous to QWIPs but claim to address issues through the mechanism of zero-dimensional quantum confinement. Multiple quantum dot material systems exhibit type-II band alignment, such as GaAs quantum dots on InAs and InSb quantum dots on InAs. Both material systems exhibit comparable lattice mismatches of around 7%, resulting in the generation of self-assembled quantum dots following the critical thickness of GaAs or InSb deposition. The confinement within the quantum dots is contingent upon their size and composition; hence, the associated interband electronic transition can be adjusted by manipulating the dot size and composition [48]. Following the reproducible and controllable epitaxial growth of self-organized quantum dots in Stranski–Krastanov (SK) mode and their fascinating physical attributes, growing interest in quantum dot physics has commenced [49]. In the Stranski–Krastanov growth mode, the strained layer undergoes a transition from the planar growth mode to 3D island formation after the critical thickness (2c) has been grown. This reduces the total free energy of the system, which is a sum of interface, bulk, and surface energies. The abrupt 2D–3D transition occurs within 0.2 monolayers (MLs) after the critical thickness, showing a rapid rise in the density of the quantum dots. This strain mediated-formation of quantum dots on a planar, strained “wetting layer” is observed across different strained material systems such as InAs/GaAs [50], InGaAs/GaAs, InAs/InP, Ge/Si, and so on.

Comprehensive studies have been published in the literature [51,52]. Stangl et al. [53] give an excellent overview on the structural properties of self-organized semiconductor nanostructures. Placidi et al. [51] mention that in heteroepitaxy of InAs/GaAs(001), the strained growing layer remains planar up to a characteristic coverage (critical thickness), above which three-dimensional (3D) islands form. Such a growth mode transition is the most distinctive aspect of the InAs/GaAs(001) system and is at the basis of the formation of self-assembled quantum dots. Although of the Stranski–Krastanov type, such a transition has a more complex evolution from its initial stage. Kratzer et al. [52] found by applying STM and theoretical calculations a shape transition in InAs islands grown by Molecular Beam Epitaxy MBE) and explained that by a thermodynamic driving force for developing a domelike shape on top of a flat base as the island grows larger.

The potential advantages of QDIPs compared to QWIPs include the following: (1) capability to absorb ordinarily incoming light due to three-dimensional confinement, thus obviating the necessity for specialized light coupling techniques like gratings, (2) diminished reliance of the carrier distribution on temperature, and (3) carrier lifetimes are 10 to 100 times greater than those of QWIPs, resulting in a reduced dark current.

Multiple research groups, e.g., [54,55], have indicated that QDIPs will substantially surpass QWIPs, establishing themselves as a crucial technology for infrared detection. Currently, QDIPs are starting to surpass QWIPs by exhibiting reduced dark current and elevated operating temperatures.

Due to the confinement of carriers in all three dimensions, if the energy separation between the two states exceeds the longitudinal optical (LO) phonon energies, carriers must absorb several phonons to transition to the excited state. A “phonon bottleneck”, characterized by a reduction in carrier relaxation rates, is anticipated in zero-dimensional semiconductor systems exhibiting a discrete density of states [54,56]. This diminishes the efficacy of the scattering mechanisms and hence significantly lowers the dark current relative to QWIP devices by prolonging the carrier lifetimes in the excited state of the quantum dot. Theory and tests have confirmed that quantum dots exhibit significantly longer carrier lifetimes, reaching up to 100 ps [57], in contrast to bulk materials or quantum wells, which are restricted to approximately 1–5 ps.

Quantum well infrared photodetectors (QWIPs)

Quantum wells are ultra-thin sheets of one semiconductor material “sandwiched” between two layers (or two 3D bulks) of another material. Carriers are confined within this plane, though they may move about with that plane.

Enhancing object recognition in the thermal infrared (IR) spectrum can be achieved by capturing radiation signals across multiple spectral intervals. IR imagers that offer on-demand spectral tuning are poised to become a critical element in systems governed by Artificial Intelligence. A notable advancement in thermal imaging is the creation of dual-band focal plane arrays (FPAs), specifically functioning within both MWIR and LWIR atmospheric windows [58,59]. This innovation greatly enhances the visibility of objects and is anticipated to improve the recognition and identification of object features across varying temperatures. The tuning between mid-wave infrared (MWIR, 3–5 µm) and long-wave infrared (LWIR, 8–14 µm) spectral regions offers notable contrast differences, rendering it highly suitable for the implementation of image fusion algorithms in object recognition applications [60]. Intersubband transition-based quantum well infrared photodetector (QWIP) technology utilizing group III-V heterostructures has garnered significant interest over the years for thermal imaging applications in both the MWIR and LWIR spectral regions. Despite the rapid advancement of other technologies, high-end applications continue to prioritize QWIP detectors because of their numerous advantages over leading competitors, such as interband HgCdTe and GaSb-based superlattice detectors. The advantages include advanced large-format GaAs technologies, excellent uniformity, consistent performance enhancement with temperature reduction for low-background scenes, and significant radiation hardness [61].

Quantum well infrared photodetectors (QWIPs) are crucial for long-wave infrared (LWIR) photon-dense systems [59], including medical imaging [62], gas detection [63], and surveillance applications [64]. Nevertheless, they exhibit low quantum efficiency, elevated dark current, an absence of typical incidence absorption, and necessitate cryogenic operating temperatures [49].

Quantum dots-in-a-well (DWELL)

A hybrid structure that combines features of the QWIP and the QDIP, referred to as the dot-in-a-well or DWELL, was introduced several years ago. This configuration has shown multiple advantages, including a multispectral response that is dependent on bias [65]. The active part of quantum dots-in-a-well (DWELL) comprises InAs quantum dots (QDs) embedded within an InGaAs quantum well, representing a hybridization of conventional quantum well infrared photodetectors (QWIPs) and quantum dot infrared photodetectors (QDIPs). The DWELL structure offers advantages including a multispectral response characterized by bias-dependent spectral tunability and reproducible control of the operating wavelength, similarly to a QWIP. Additionally, it features low dark current and a normal incidence operation akin to a QDIP [66]. The multispectral response arises from various transition energies, including dot to dot, dot to well, and dot to continuum. Spectral tunability is achieved through band bending, which is influenced by the application of bias voltage that alters the transition energies [66,67].

Similarly to QDIPs, DWELL detectors operate at normal incidence without the need for gratings or optocouplers, while also providing reproducible “dial-in recipes” for precise control over the operating wavelength, akin to QWIPs. Femtosecond spectroscopy revealed long carrier lifetimes in DWELL heterostructures, indicating their potential for high-temperature operation. Additionally, DWELL detectors exhibit bias-tunability and multicolor operation across the mid-wave infrared (3–5 µm), long-wave infrared (LWIR, 8–12 µm), and very long-wave infrared (>14 µm) ranges [66,68]. DWELL-based LWIR 320 × 256 focal plane arrays that operate at liquid nitrogen temperatures have been developed [65]. The first two-color, co-located quantum dot-based imaging system that can be used to take multicolor images using a single FPA was reported by Varley and colleagues [69]. Shenoi et al. [70] changed the DWELL structure by incorporating quantum dots (QDs) into a quantum well (QW) structure, subsequently embedding this hybrid configuration into another QW, termed double DWELL or DDWELL. This novel construction offers the benefit of reduced strain in the heterostructure, resulting in enhanced operational temperatures while preserving minimal dark current [71].

One potential issue with these detectors is their low quantum efficiency, resulting in reduced responsivity and detectivity. Reduced dark current, minimal electron–phonon scattering, and extended carrier lifetime can be achieved with quantum dot-based devices [48,72]. Diverse growth strategies have been suggested to enhance the optical and structural characteristics of quantum dots (QDs), including Stranski–Krastanov, droplet epitaxy mode, atomic layer epitaxy, submonolayer (SML) mode, quasi-monolayer, and organo-metallic vapor-phase epitaxy (OMVPE).

A capping layer on quantum dots is necessary to ensure uniformity in shape and size. The capping layer or well layer can be deposited using two methods: the analog alloy capping layer (AACL) and the digital alloy capping layer (DACL) approach [73,74]. The AACL approach utilizes a single thick well layer minimizing strain on both sides of the well layer interfaces by achieving a minimal lattice mismatch at one of the interfaces of the well layer. The DACL with SPS concept has addressed challenges related to the AACL. In contrast, the DACL approach involves dividing the bottom or top well layer, based on its thickness, into equal sub-parts composed of the same material but differing in composition [75]. A comprehensive examination of the DACL approach is presented in Refs. [76,77].

Type-II superlattice (T2SLs) photodetectors

Particular emphasis is given on HgCdTe ternary alloys on silicon, type-II superlattices, uncooled thermal bolometers, and innovative uncooled micromechanical cantilever detectors. Despite formidable competition from other technologies and slower-than-anticipated advancements, HgCdTe is unlikely to face significant challenges in high-performance applications, particularly those necessitating multispectral capabilities and rapid reaction. Nonetheless, nonuniformity is a significant issue for LWIR and VLWIR HgCdTe detectors. In this context, the type-II superlattice system is anticipated to serve as an alternative to HgCdTe in the long wavelength spectrum region. In established uncooled imaging, VOx microbolometer arrays are the predominant technology. Despite significant competition from other technologies and slower-than-anticipated advancements, HgCdTe is improbable to face substantial challenges in high-performance applications, particularly those necessitating multispectral capabilities and rapid reaction. Nonetheless, nonuniformity is a significant issue for LWIR and VLWIR HgCdTe detectors. HgCdTe is likely not the ideal choice for applications necessitating operation in the LWIR spectrum and dual-color MWIR/LWIR/VLWIR bands [78].

In a work of Sai-Halasz et al. [79] approximately 50 years ago, they developed and theoretically studied a novel bilayer semiconductor superlattice whereby the lower conduction band (CB) edge is in one material, while the upper valence band (VB) edge is situated in the other. The essential characteristic that facilitated the type-II superlattice concept was the broken-gap band alignment between InAs and GaSb. In this type of superlattice, the wavefunctions of the lowest conduction sub-band and the highest valence sub-band are confined to two distinct regions (two spatially localized semiconductors). This gives the opportunity to tune the two bands (Cd and VD) independently [80]. It is evident that InAs and GaSb exhibit a nearly lattice-matched material system that provides significant versatility in the construction of infrared optoelectronic devices. Currently, III-V type-II superlattice (T2SL) detector technology is undergoing significant research as a potential replacement to HgCdTe. Innovative concepts in detector design have elevated the status of T2SLs in infrared materials detection technology. T2SLs are particularly advantageous in the design of unipolar barriers [81].

Barrier designs demonstrate improved performance characterized by reduced dark current and enhanced operating temperature [82]. Various superlattice-based detectors and focal plane arrays (FPAs) utilizing MW/LW, LW/LW, and SW/MW/LW configurations have been published [83,84].

The Type-II InAs/GaInSb superlattice structure is a novel alternative infrared material system, demonstrating significant potential for LWIR/VLWIR spectral ranges, with performance akin to HgCdTe at equivalent cut-off wavelengths [78,85,86]. In order to achieve cut-off wavelengths within the 8-to-12 µm range, InAs/GaInSb superlattice p–i–n photodiodes are fabricated, featuring an indium molar fraction in the ternary GaInSb layers approximately at 20% [87]. Type-II InAs/GaInSb detectors have experienced significant advancements in recent years [88,89]. A simultaneous dual-band FPA has been built in the MWIR [90,91] and large-format focal plane arrays (FPAs) have been shown in research facilities in the long-wave infrared (LWIR) spectrum [59,92].

The Type-II InAs/GaSb superlattice structure, fabricated on GaSb supports, presents a compelling alternative to the leading mercury cadmium telluride (MCT) photodiodes and quantum well IR photoconductors, owing to its superior spatial uniformity and enhanced capability to precisely adjust the cut-off wavelength. Despite the advanced development of industry-standard MCT detectors, it has been acknowledged that the material exhibits significant limitations for specific applications [93].

T2SL photodiodes are generally developed utilizing a P-i-N architecture, featuring an accidentally doped intrinsic region (ν or π) situated between severely doped p-type and n-type materials with greater bandgaps that are lattice-matched. The diminished minority carrier concentration in the high bandgap layers results in a reduced diffusion current, increased RoA, and enhanced detectivity. According to Rogalski et al. [81], the primary technological issue in the manufacture of T2SL detectors is the creation of sufficiently thick, high-quality active areas to achieve acceptable quantum efficiency. Quantum efficiency over 50% is achieved due to a substantial absorption area (>5 μm) in both LWIR P-i-N photodiodes and barrier detectors.

Submonolayer Quantum Dots (SML QDs)

Submonolayer quantum dots (SML QDs), proposed as an alternate method for quantum dot creation [94], has lately garnered attention due to its potential of producing highly dense and uniform quantum dots [95,96]. The formation entails the deposition of a short-period InAs/GaAs superlattice on a GaAs (100) substrate, with an effective InAs thickness that is under 1 monolayer (ML) and an appropriate number of repetitions, leading to the creation of nanometer-scale (In,Ga)As quantum dots of a non-SK class [97]. Since InAs possesses a larger lattice constant than GaAs, the thin GaAs spacer layer that partially covers the InAs monolayer is subjected to significant tensile strain in the regions where it overlaps with the InAs material. The corresponding local rise in lattice constant creates advantageous sites for the nucleation of InAs in the subsequent layer, ideally resulting in a vertical alignment of the SML InAs material. The vertical separation between InAs depositions is significantly less than the wavefunction of a confined carrier; hence, the wavefunction encompasses the entire InAs stack, resulting in the InAs SML stack forming an InGaAs quantum dot within a GaAs matrix. Such SML QDs do not form a wetting layer, and their density is significantly higher (≥10^12^ cm^−2^) [98,99] than that of SK quantum dots [100].

Ledentsov and Bimberg [96] asserted that nanocomposites exhibit a significant alteration in the optical characteristics of active media in comparison to the properties of the individual constituent materials. The utilization of nanocomposite semiconductor materials facilitates the expansion of the wavelength range of optoelectronic devices on GaAs substrates to the blue-green spectral range (450–550 nm) through type-II GaAs–AlAs quantum dots, while also extending to telecom wavelengths (>1.3 μm) using InAs–GaAs quantum dots. Infrared photodetectors utilizing multi-stack SML quantum dots exhibit enhanced device performance, including reduced dark current density, increased responsivity, and improved detectivity relative to traditional S-K quantum dot infrared photodetectors. Furthermore, the performance of SML QDIPs is significantly enhanced by the construction of an appropriate AlGaAs potential barrier, which facilitates superior quantum confinement of carriers [101].

Other concepts and materials

Recently, Halenkovic et al. [102] have synthesized amorphous chalcogenide films within the Ge-Sb-Se-Te (GSST) system, demonstrating extensive infrared transparency alongside elevated (non)-linear refractive indices. These characteristics render these materials appropriate for prospective applications in mid-infrared electronics, including optical switches and sensors.

Ma et al. [103] proposed a novel IR absorber (a perfect absorber as they claim) consisting of a continuous Au film, a FR-4 spacer, a multilayer Dysprosium-doped cadmium oxide (CdO:Dy) film, and a gold ring array arranged from bottom to top. In multilayer CdO:Dy materials, various epsilon-near-zero (ENZ) modes are stimulated by the surface plasmon mode of the gold ring. The ENZ resonance wavelengths, influenced by the doping concentration and the layer count of CdO:Dy material, dictate the multifrequency or broadband absorption characteristics of the infrared absorber. Four narrowband peaks are generated at 1311 nm, 1560 nm, 1732 nm, and 1870 nm, with absorbance of more than 92.6%. The same group has also developed a dynamically tunable and a switchable ideal infrared absorber is proposed, consisting of a gold ring array, an indium tin oxide (ITO) layer, an alumina (Al_2_O_3_) spacer, and a continuous gold (Au) film from top to bottom. The epsilon-near-zero (ENZ) mode in the indium tin oxide (ITO) layer is stimulated by the localized surface plasmon resonance (LSPR) mode of the gold ring. The electrically controllable absorption switch feature endows this absorber to a material with potential applications in selective thermal emitters, infrared sensors, infrared stealth, beam focusing, and other domains [104].

## 4. Applications of IR Sensors

### 4.1. Directional Sensors

IR sensors are extensively utilized as proximity detection devices and for avoidance of obstacles in robots. They provide reduced costs and expedited reaction times compared to ultrasonic (US) sensors. Nonetheless, due to their non-linear characteristics and reliance on the reflectance of adjacent objects, measurements derived from the intensity of back-scattered infrared light are highly inaccurate for range applications. Consequently, environment maps generated with this type of sensor exhibit worse quality, and infrared sensors are predominantly utilized as proximity monitors in mobile robots [105].

### 4.2. Thermal Photodetectors, Pyroelectric Sensors, Thermopiles, Bolometers

A thermopile comprises one or more thermocouples connected in series, in a certain arrangement such that the hot junctions create a tiny circle, while the cold junctions are kept at the ambient temperature of the surroundings. Advanced thin film thermopiles attain response times between 10 and 15 milliseconds. Thermopiles amplify the output signal strength and are the optimal selection for broadband thermometers. Ambient temperature correction is necessary for the utilization of thermopile detectors. A thermostatically regulated thermometer casing must be employed to mitigate ambient temperature variations for low-temperature applications.

A bolometer is an instrument that quantifies radiant heat through a medium with electrical resistance that varies with temperature. Bolometers are basically resistance thermometers configured to detect radiation and “are thousand times more sensitive to radiant heat than the thermopile, and capable of indicating a change of temperature as minute as 1–100,000th of a single Centigrade degree” was already reported in *Nature* in 1881 [106]. A sensing element utilizing a thermistor, metal film, or metal wire transducer is sometimes referred to as a bolometer. Currently, the majority of bolometers employ semiconductor or superconductor absorptive components and function at cryogenic temperatures, allowing for markedly enhanced sensitivity. A microbolometer is a specialized form of bolometer utilized as a detector in thermal imaging cameras. Microbolometers surpass thermopiles regarding image quality. When properly calibrated, they often exhibit reduced noise, yielding more detailed images essential for automatic identification. Microbolometers possess also some drawbacks relative to thermopiles; they necessitate regular calibration to eliminate noise from images, and certain models may not function across all temperature ranges. Thermopiles have the potential to serve as an effective thermal imaging instrument, provided there are enhancements in noise reduction and pixel resolution, particularly if their price remains affordable.

The identification of defects in process industries utilizing metallic equipment and structures is a critical maintenance procedure. The significance of proper detection techniques is underscored by the limitations that result in restricted examination of materials while the process is running. The recent advancement in thermographic defect detection addresses the issue effectively. The thermographic technique is applicable for in situ defect detection, particularly as equipment and components in chemical engineering processes operate at elevated temperatures. The process operation can continue without interruption for the detection of defects in components such as vessels and pipes using thermography, a method that offers potential savings in both costs and time. A low-cost thermographic device for detecting grooves in metal plates has been developed by Kim et al. [107], and its performance is evaluated by comparing experimental results with numerically solved temperature profiles. The sensor module is equipped with five infrared temperature sensors arranged in a linear configuration, along with signal amplification circuits. The experimental measurements conducted under two distinct temperature distribution settings in the sample plate reveal the position of a hidden groove, corroborated by the numerical solution of the distribution.

The IR detector’s sensitivity to non-idealities and fluctuations in ambient temperature Ta, and the temperature of the substrate Ts, necessitates effective thermal shielding, hence elevating its cost. The detector’s sensitivity to thermal noise and variations may be mitigated by co-integrating the infrared detector with integrated Peltier devices to dynamically regulate the device temperature. For a thermopile-based infrared detector, co-integration is straightforward, as both the Peltier element and the thermopile are composed of the same materials [108].

Samkaran et al. [109] illustrated the efficacy of visible–near-infrared and thermal imaging for the identification of Huanglongbing (HLB) disease in citrus trees. Spectral reflectance data in the visible–near-infrared (440–900 nm) and thermal infrared ranges were obtained from individual healthy and infected trees. Data analysis indicated that the mean reflectance values of healthy trees in the visible spectrum were inferior to those in the near-infrared spectrum, whereas the reverse was true for HLB-infected plants. Furthermore, the 560 nm, 710 nm, and heat bands exhibited the highest degree of separation between healthy and HLB-infected trees of all assessed visible-infrared bands.

A rising number of applications necessitate very sensitive photodetectors in the mid-infrared spectrum that do not require cooling. Thermal photodetectors, including bolometers, have become the preferred technology due to their lack of cooling requirements [110].

Pyroelectric detectors are capacitor-like structures where a pyroelectric crystalline material like lithium tantalate, lithium niobate, triglycine sulfate, or barium titanate is sandwiched between two metal electrodes. They have significant benefits regarding cost and operational simplicity compared to cooled photon detectors for long-wavelength infrared light. Pyroelectric detectors are widely documented for their minimal power consumption, economical production, rapid reaction, and comparatively good sensitivity throughout an extensive temperature range and broad spectral bandwidth [3,111,112].

Wen and colleagues [113] developed a flame intensity sensor utilizing the resistive and memory characteristics of a spintronic memristor to assess flame intensity in which the impact of ambient temperature on sensor performance can be mitigated by utilizing the resistive characteristics of spintronic memristors. The experimental results indicate that for a flame combustion temperature change rate of 1 °C/s, the sensor measurement range is 0–3.1 mW/cm^2^, with a sensitivity of 4.516 kΩ/(mW/cm^2^). The sensor facilitates continuous multiple measurements of flame intensity and possesses an extended measurement range.

In various sensor applications, such as infrared detectors [114,115], thermoelectric gas sensors [116], and thermal flow sensors as described in [117,118], thin-film thermopiles are employed as high-precision thermometers. Temperature differences between two points are measured by thermopiles. According to the Seebeck effect [119], the produced thermopower is proportional to the temperature differential between the two material contacts (junctions). For thermal isolation, the hot junction in infrared detectors and flow sensors is positioned near a heater or absorbing region on a membrane or free-standing bridge. The bulk material serving as a heat sink is where the cold junction is positioned [120].

### 4.3. Imaging-Thermography and Tracking

**Figure 8:**
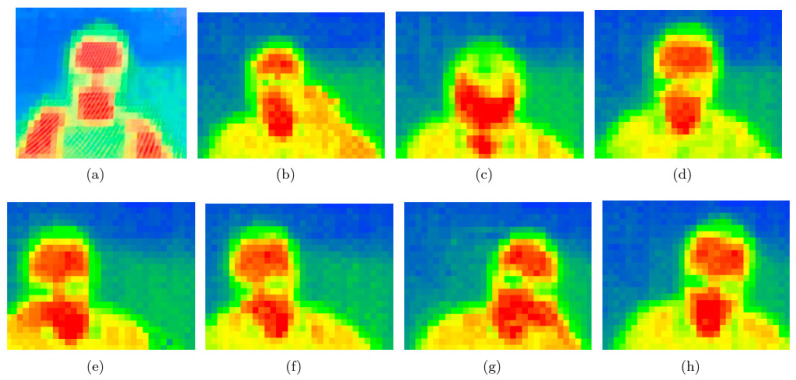
Output image of each detected sitting posture. (**a**) Lean back, (**b**) head up (e.g., neck rest), (**c**) head down (e.g., writing), (**d**) normal sitting, (**e**) lean left with arm support, (**f**) lean left without arm support, (**g**) lean right with arm support, (**h**) lean right without arm support. (Reproduced from [123] with permission).

Their implementation of Internet of Things (IoT) and wearable sensor technology [121] in the workplace leads to the concept of smart offices, defined as environments that proactively yet judiciously assist individuals in their regular tasks. Currently, numerous office employees are experiencing a condition of subhealth due to prolonged sitting and an overly sedentary lifestyle [122]. Detecting employment status may foster improved work habits and diminish the risk of obesity, diabetes, and cardiovascular disease. Ma and coworkers [123] have developed a recognition method that can discreetly monitor employees’ actions while respecting privacy in a kind of smart office. To assess the system’s performance, a functional scenario was established, and the subjects’ activities were annotated by video analysis as can be seen in (Figure 8).

In an application for automatic door opening, Venkataramanan et al. [124] used a passive IR sensor for the contactless measurement of the body temperature of humans that allowed the entrance of only individuals with temperatures less than a threshold. Tracking sensors with commercial passive infrared (PIR) sensors were prepared and tested by Ngamakeur et al. [125] for the localization and tracking of moving people.

Additive manufacturing of metals and ceramics is a very promising technique that facilitates the economic production of intricate workpieces in limited quantities. Nevertheless, in safety-critical domains, it is essential to implement solutions that ensure compliance with stringent in situ and ex situ testing and quality standards [126]. Laser powder bed fusion is susceptible to defect generation throughout the fabrication process, necessitating a solution to facilitate its widespread deployment. A potential solution to this issue is employing in situ thermographic monitoring for defect identification. Scheuschner et al. [127] employed two distinct wavelengths (near-IR (NIR) and short-wave-IR (SWIR)) to gather data on emerging flaws via optical bandpass filters with a short-wave infrared (SWIR) camera, which has a detection range that overlaps with silicon-based sensors.

### 4.4. Environmental and Gas Sensors Applications

Devices with almost perfect absorption characteristics are in high demand for many applications, including heat storage, photovoltaic solar cells, and photodetectors (PDs). Advanced infrared photo-absorbing devices are sought after in areas such as spectroscopy, imaging, remote sensing, and communication. Infrared molecular spectroscopy, utilizing the distinctive vibrational modes of molecules within this spectral range, is employed in various applications, including gas concentration measurements for industrial process control, environmental monitoring, and the detection of diverse explosives for security and safety systems [128,129]. Avrahamy and colleagues [130] proposed and developed a novel concept for Mid-IR spectroscopy that leverages the exceptional sensitivity of the spectral absorption of its PD MM pixel to the azimuthal incidence angle, thereby introducing an additional dimension for encoding and decoding spectral information, which offers significant advantages over traditional designs, including those utilizing diffraction gratings. The suggested wavelength scanning mechanism, which employs azimuth-incidence angle adjustments to modulate optical absorption in a specifically engineered ultra-thin metamaterial, along with a novel spectrometer concept derived from this mechanism, has the potential to drive the creation of entirely new devices that could supplant various existing complex and cumbersome optical components and configurations, while surpassing their performance. This can aid many MWIR-range applications across multiple fields, including chemistry, biology, medicine, etc.

Argirusis and coworkers [131] have investigated the effectiveness of combining Internet of Things (IoT) technology with machine learning algorithms to categorize refrigerant gases, namely R32 and R134a. The method employed a variety of passive IR gas sensors linked through an IoT network, enabling the acquisition of an extensive dataset under regulated settings. This dataset was subsequently utilized to train other machine learning models, including Random Forests, Support Vector Machines (SVMs), and Neural Networks (MLP). The Random Forest algorithm exhibited exceptional performance owing to its robustness, precision, and computing economy. The combination of IoT with machine learning for refrigerant gas categorization offers a viable option for real-time environmental surveillance and safety applications.

Gas detection is a major application of IR sensors in addition to expensive high-definition FTIR spectrometers that may be prohibitive for everyday industrial applications; one may also utilize the low-cost, non-dispersive infrared (NDIR) spectrometer. The sensitivity and high efficiency of Fourier transform infrared (FT-IR) spectrometers, along with the high optical efficiency of mid-infrared fiber optic materials and sensors, have created an optimal instrument for remote sampling [132]. Fulladosa et al. [133] examined and compared the efficacy of bench-top (FT-NIR) and low-cost near-infrared (LC-NIR) spectrometers in quantifying salt content and texture in canned tuna. The distribution of salt content was examined utilizing hyperspectral imaging (HSI) and computed tomography as well for comparison reasons. The data analysis led to prediction errors for salt content of 0.10%, 0.22%, and 0.22% for FT-NIR, LC-NIR, and HSI, respectively. Low-cost sensors may provide an effective means to standardize production and facilitate accurate nutritional labeling.

The calibration of thermal cameras differs from that of optical cameras, typically employing ground-based calibration targets with established temperatures, which are measured using portable infrared thermometers [134] for temperature adjustment. Precise calibration ensures data reliability and mitigates the influence of mistakes on modeling and interpretation. It is imperative to conduct accurate calibration prior to data extraction and analysis [4].

Martins et al. [135] have presented a forest fire detection solution utilizing small autonomous aerial vehicles that employ optical data from low-cost sensors, namely one operating in the visible spectrum and another in the near-infrared spectrum, integrated into an ignition detection architecture and system.

Unmanned aerial vehicles (UAVs) equipped with remote sensors are extensively utilized in the domains of architectural, civil, and environmental engineering. UAVs are utilized for the monitoring of civil infrastructure to assess the causes of failure in forensic engineering. Applications have been identified in geotechnical engineering, particularly concerning failures like slope failures and ground deformation, which are critical for infrastructure safety and maintenance [136,137]. Additional uses of infrared sensors can be found in structural engineering and water infrastructure engineering. Hyperspectral cameras have been utilized to monitor pollution sources released from various origins, such as domestic sewage, terrestrial inputs, agricultural runoff, algal outputs, and industrial wastewater sources [138]. The data acquired through the UAV photogrammetry method exhibit lower accuracy in comparison to in situ or on-site measurements. This discrepancy arises from the inherent technical limitations of the sensor and the conversion process from image data to actual data [6].

Environmental sensor networks (ESNs) offer novel prospects for using sensors to observe environmental alterations. Environmental sensor networks (ESNs) can enhance our comprehension of environmental phenomena and inform natural resource management (NRM), such as assessing the effects of climate change and grazing on tree regeneration across multiple seasons, monitoring weed invasions, evaluating grazing impacts on indigenous groundcover, and tracking cattle movements. Environmental sensor networks often denote sensing conducted in immediate vicinity to the target, in contrast to remote sensors that detect at considerable distances from the target. In environmental sciences, sensors encompass digital cameras, visible to near-infrared spectrometers, and soil moisture sensors, among others [139].

**Figure 9:**
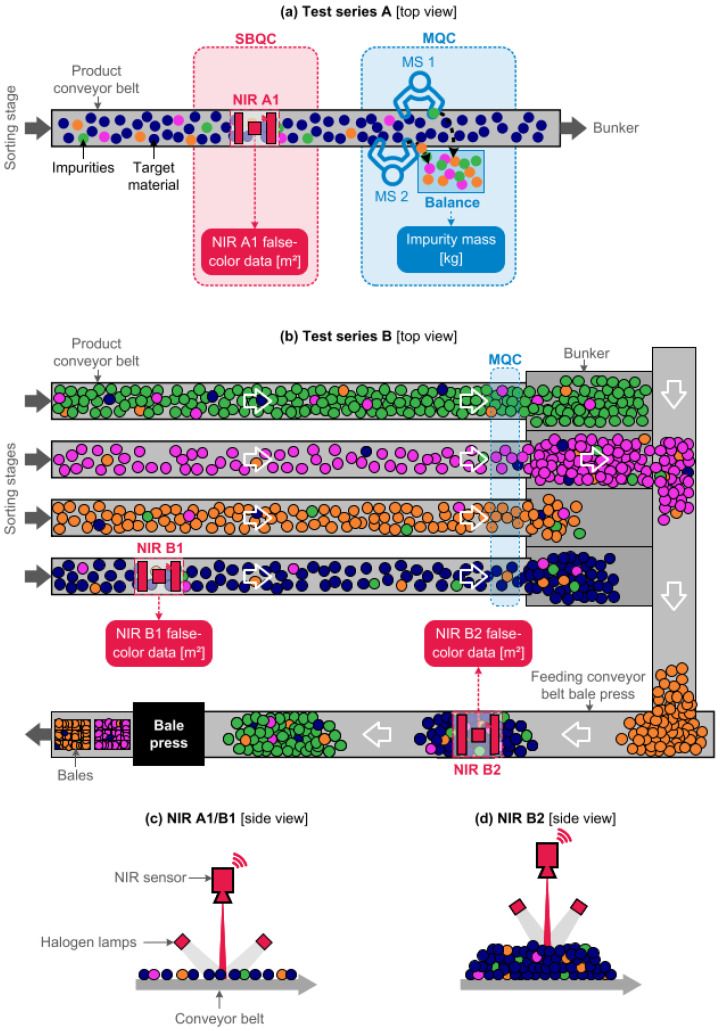
Experimental configuration of (**a**) test series A and (**b**) test series B, along with the NIR sensor arrangement and material flow depiction at (**c**) sensor position P1 [PET tray product conveyor belt, monolayer material flow depiction] and (**d**) sensor position P2 [feeding conveyor belt bale press, multilayered material flow depiction]. (Reproduced from [142] with permission).

Kroell et al. [140] have established the technological viability of forecasting mass-based material flow compositions from NIR-derived false-color data utilizing machine learning (ML) that allows for predicting the mass-based material flow compositions of binary post-consumer plastic packaging mixtures uncertainty of ±2.0 wt% at a technical laboratory scale [141]. Recently, the technological viability of NIR-based quality control for plastic pre-concentrates at a plant scale (Figure 9) has been demonstrated by the same group [142].

Focusing on the vis-NIR wavelengths, Hu, C. [143] offered insights into the detectability and discrimination of various forms of marine debris from other floating materials. This assessment was based on published end-member spectra, sensor sensitivity, simulation experiments, and spectral analyses of Sentinel-2 data for demonstration purposes, leading to recommendations regarding sensor design and algorithms for vis-NIR remote sensing of marine debris. The application of SWIR wavelengths is also examined.

Munyati [144] reports on the use of Landsat-9 images obtained with the Thermal Infrared Sensor-2 (TIRS-2) band 10 (B10) for the localization of thermal phenomena in urban green spaces (UGSs). The cooling effect (CE) of underground gas storage (UGS) has rarely been shown in small towns situated in dry and hot regions where its necessity is as severe. Measuring the effect would possibly be beneficial for municipal planning by enhancing the welfare of urban residents [145] and contributing to climate change mitigation, as vegetation reduces atmospheric carbon dioxide (CO_2_) concentrations [146].

The Silica Index (SI) derived from thermal infrared bands is an efficient method for detecting sandy desertification. The Sustainable Development Goals Science Satellite 1 (SDGSAT-1) features a thermal infrared sensor with superior spatial resolution (30 m) and an extensive swath (300 km), enhancing global thermal infrared imagery through three thermal infrared bands, thereby providing significant potential for large-scale desertification mapping. The suggested Relative Normalized Silica Index (RNSI) delineates sandy desertification areas utilizing SDGSAT-1 thermal infrared radiance at approximately 9.35 μm and 10.73 μm, grounded in the spectral emissivity properties of sandy terrain. The results indicate that the RNSI could successfully eliminate the impact of land surface temperature variations in the original SI, hence ensuring robust spatial consistency in the creation of large-scale SI datasets. The suggested composite RNSI can accurately categorize various sandy desertification areas, with an overall accuracy of 85.7% [147].

The increasing scarcity of freshwater poses a significant challenge to food security and sustainability, as agriculture is the largest yearly consumer of water. Precision agriculture (PA) has made substantial advancements in water management and crop productivity through technical innovations in software and hardware [148,149]. Therefore, Jiménez et al. [150] proposed an IR-based technology for agricultural irrigation management by monitoring the crop canopy temperatures. Canopy temperatures can be assessed using radiometers calibrated for thermal infrared wavelengths (8 to 14 μm), rendering them non-contact infrared thermometers. These instruments may quantify an area from a few cm^2^ to many km^2^, depending on the platform, which is generally classified as ground-based, aerial, or satellite.

The warming planet is significantly affected by the rising frequency, intensity, and duration of heatwave occurrences, which result in considerable societal and environmental harm and ramifications from local to global levels. Overcoming the constraints of site observations in precise geographical extent identification and extensive monitoring is considered necessary. Hu and coworkers [151] demonstrated that remote sensing datasets (satellite-based surface temperature datasets) are capable of providing the continuous whole-Earth coverage of heat wave changes, and they would play more important roles in preventing or mitigating the impacts of extreme heat events on people and natural environment.

### 4.5. Medical Applications

The World Health Organization (WHO) report [152] indicates that air pollution was responsible for approximately 4.2 million deaths in 2016, while household air pollution accounted for around 3.6 million deaths during the same period. Furthermore, risk factors for non-communicable diseases (NCDs) contribute to an estimated one-quarter (24%) of all adult deaths due to heart disease, 25% due to stroke, 43% due to chronic obstructive pulmonary disease, and 29% due to lung cancer. In 2019, air pollution was identified as the most significant threat to health [152]. Given the aforementioned problems, air pollution is currently the biggest environmental health risk on the globe. Indoor air quality is crucial, given that individuals spend a significant amount of time in indoor environments such as homes, hospitals, and schools. These settings are often monitored for various hazardous gases, including CO_2_ [153], CO, benzene, toluene, and volatile organic compounds (VOCs), along with humidity levels. The long-term effects of these factors on humans include respiratory infections, lung cancer, and heart diseases. Consequently, the detection of air pollution in both indoor and outdoor environments is of equal significance [154].

**Figure 10:**
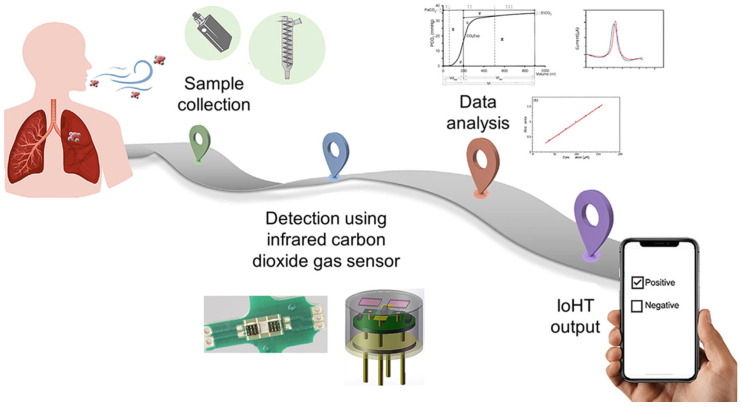
Architecture for monitoring and diagnosing respiratory illnesses utilizing optimal infrared CO_2_ gas sensors with easily obtained exhaled air. The sensing mechanism is anticipated to deliver immediate readouts for data analysis while ensuring secure and efficient data storage through IoT networking, readily accessible in clinical and home environments. (Reproduced from [155] with permission).

Chronic respiratory diseases represent the primary cause of morbidity across all age groups, accounting for approximately 3 million deaths globally. Exhaled breath is the most readily available sample for diagnosing respiratory diseases, easily produced by humans. Non-invasive diagnosis of respiratory diseases via exhaled breath provides simple clinical monitoring, minimal infection risk, and a highly repeatable methodology. Carbon dioxide (CO_2_) serves as a promising biomarker, since its varying expiration levels provide the assessment of systemic metabolism, ventilation, and pulmonary dysfunction (Figure 10), hence offering diagnostic insights into respiratory and pulmonary disorders [155].

Fiber-optic evanescent wave spectroscopy (FEWS) is a technique that facilitates the measurement of absorption spectra within an optically closed system. Infrared (IR) fiber sensors have numerous potential applications in environmental investigations, process control, chemical synthesis and analysis, and life sciences, e.g., for bio-medical sensing and clinical purposes like blood coagulation monitoring, and tissue or biofluid on-line diagnostics. IR fiber-optic sensors can also be utilized for needle biopsy or catheterization. In minimally invasive medicine and open surgery, IR-endoscopy (spectroscopic data combined with a visible image) may serve as a novel instrument for in situ diagnostics and real-time monitoring [156].

**Figure 11:**
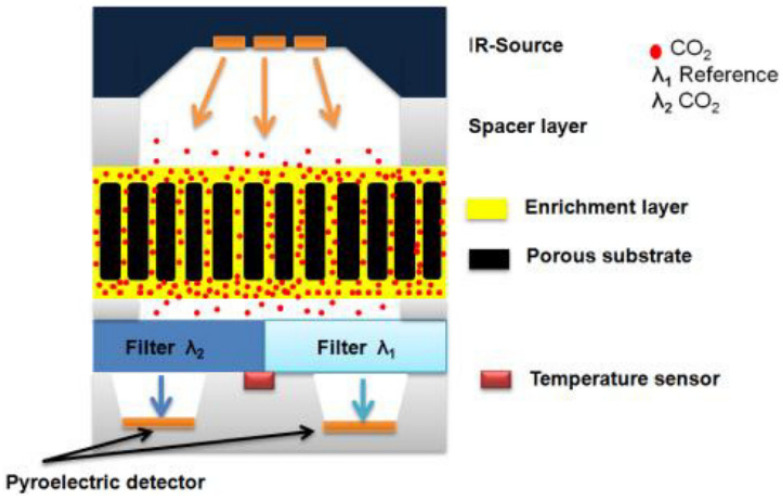
Schematic diagram of the miniaturized IR-based CO_2_ sensor comprising an enrichment layer (target distance between the emitter and the detector ≤ 5 mm). (Reproduced from [157] with permission).

Moumen and coworkers [157] have developed a miniaturized hybrid NDIR/chemical CO_2_ sensor by combining both IR and chemical technology. The sensitivity of the miniaturized CO_2_ sensor is increased by applying a CO_2_ sensitive film as an enrichment layer (Figure 11).

Thermography can be applied also in metallic materials for the non-invasive assessment of fatigue failures in them. D’Accardi et al. [158] have demonstrated the viability of conduction thermography for the identification and quantitative characterization of small fatigue cracks, examining several materials and analyzing several thin specimens. Given the established configuration utilizing low-cost devices for energy sources and sensors with minimal current values, it has been demonstrated that quantitative results regarding crack length estimation can be obtained. Additionally, this method offers advantages such as the ability to inspect surfaces without black coating and in an offline manner.

**Figure 12:**
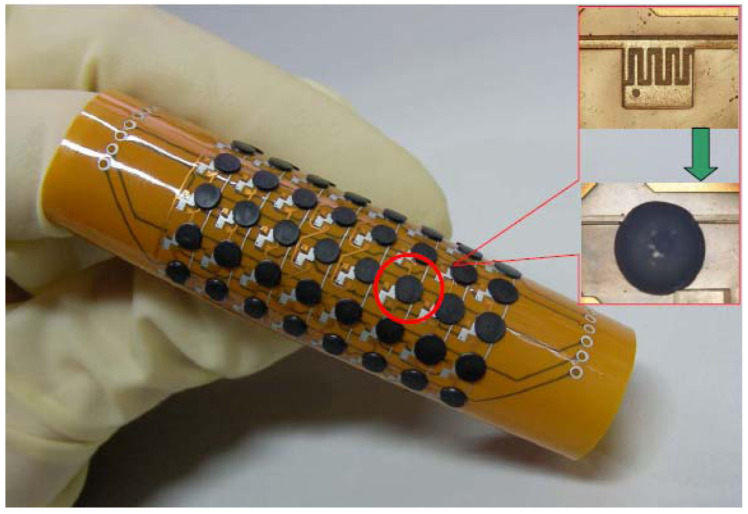
Fabricated flexible temperature sensor array. The insets show the interdigitated electrode and composites on the electrode, respectively. (Reproduced from [161] with permission).

Organic polymers, referred to as conducting polymers (CPs), have gained popularity owing to their distinctive electrical and optical properties. The material properties of conductive polymers (CPs) resemble those of certain metals and inorganic semiconductors, while also maintaining polymer attributes such as flexibility and ease of processing and synthesis, typically linked to conventional polymers. Conductive polymer sensor elements with potential applications in clinical diagnosis and surgery are being developed [159]. Thermal sensing has emerged as a valuable diagnostic instrument in applications such as thermographic imaging or infrared thermal imaging, utilized for detecting minor temperature variations associated with vascular disorders, pre-clinical breast cancer diagnosis, identification of neurological disorders, and monitoring of muscular performance [160]. Thermal sensors integrated into touch-sensitive probes or catheters can serve as surgical instruments that assist clinicians in quantifying subtle variations in tissue temperature during surgery, particularly in ablation techniques that utilize laser or RF energy for the removal or abrasion of defective tissues. Furthermore, probe-based thermal sensors can deliver real-time temperature profiles of tissues, enabling doctors to accurately regulate heat energy and avert unintended tissue injury. Shih et al. [161] constructed thermo-resistive sensor arrays by applying a graphite-PDMS composite onto flexible polyimide films (Figure 12). The sensor was engineered for application as an E-skin to facilitate haptic interaction with robots.

Dayeh et al. [162], have also fabricated 1 × 10 micromachined infrared sensor arrays on flexible polyimide substrates using semiconducting yttrium barium copper oxide, YBCO, as radiation sensitive material.

Surface plasmon resonance (SPR) sensor technology has been extensively utilized in biometrics; nevertheless, its limited detection capabilities and low sensitivity hinder the advancement of SPR biosensors, circumstances that led to a recent study by Yin et al. [163] proposing the utilization of the transition metal disulfide (TMD) material MoS_2_ to create the surface plasmon resonance (SPR) phenomenon in the near-infrared spectrum. The objective of this study was to create near-infrared sensors that can quantitatively measure cDNA concentration, addressing issues of low sensitivity and parameter crosstalk. The findings indicate that the sensitivity of the SPR sensor at infrared wavelengths is 1.69 times greater than that at visible wavelengths, and infrared waves are more appropriate as an excitation source for the SPR effect due to their stronger evanescent field.

### 4.6. Other Applications

#### 4.6.1. Energy Harvesting

The pyroelectric effect facilitates the conversion of temperature fluctuations into usable electrical energy. The dissipation of heat in our daily surroundings and the industrial sector represents a significant source of energy [164]. Sensors in industrial and institutional purposes have a lengthy history, having evolved considerably with the advent of IoT. IoT platforms utilize many sensor types for independent data collecting, processing, and dissemination, promoting intelligent ecosystems. IoT devices link to sensor networks, enabling data collection and transmission, hence improving efficiency through diverse sensor types, e.g., as infrared (IR) sensors for optical, motion, temperature, and environmental related applications [165,166].

Considering all forms of energy harvesting technologies, transformation of thermal energy into electrical energy is particularly ideal for temperature sensors owing to its high sensitivity to temperature fluctuations. At present, high-performance materials that enhance the output power of energy harvesting devices while reducing power consumption present an exciting prospect for power generation and applications in self-powered sensing systems [12].

**Figure 13:**
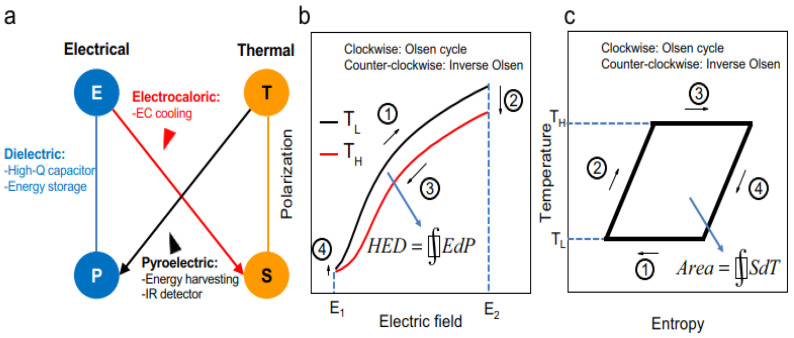
(**a**) Schematic representation illustrating the interaction between electrical and thermal properties. (**b**) Schematic diagrams illustrating the Olsen cycle for energy harvesting and electrocaloric cooling within temperature-dependent polarization–electric field curves. (**c**) Schematic diagram illustrating the Olsen cycle within the temperature–entropy plane. (Reproduced from [167] with permission).

Antiferroelectric Hf_x_Zr_1−x_O_2_ (HZO, x = 0.1–0.3) films are presented as novel Si-compatible materials for monolithic devices utilized in pyroelectric energy harvesting, electrocaloric cooling, electrostatic energy storage, and infrared sensing. The Hf_0.2_Zr_0.8_O_2_ and Hf_0.3_Zr_0.7_O_2_ films may function as pyroelectric energy harvesters utilizing the Olsen cycle (Figure 13), achieving energy densities of 11.5 and 5.7 J cm^−3^ cycle^−1^, respectively, which are 7.6 and 3.7 times more than the highest value previously documented. The electrocaloric effect (ECE) of HZO films was initially investigated, revealing maximum ΔT values of 13.4 K (at 307 K) for Hf_0.2_Zr_0.8_O_2_ and 9.8 K (at 448 K) for Hf_0.3_Zr_0.7_O_2_ [167].

**Figure 14:**
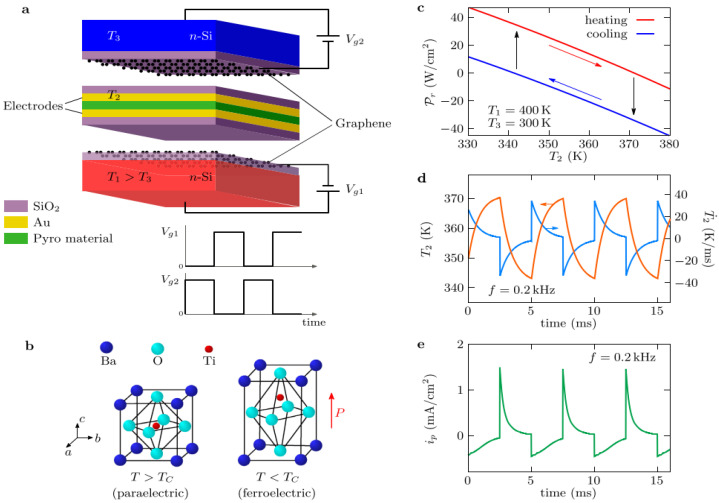
Pyroelectric converter utilizing graphene. (**a**) Schematic representation of the device, where a pyroelectric membrane (active zone) is positioned between two GFETs (thermal reservoirs) and maintained at distinct temperatures T1 (primary source) and T3 < T1 (thermal sink). The modulation of bias voltages Vg1 and Vg2 applied to the GFET gates facilitates the oscillation of the membrane temperature T2, resulting in the generation of useful power through the pyroelectric effect. The electrodes in the active zone function to extract electric charge and generate an electric field. (**b**) The crystallographic structure of BaTiO_3_ indicates the presence of a permanent polarization P in the c-direction when the temperature T is below the Curie temperature TC. (**c**) The heat flux received by the membrane during the heating and cooling steps induced by the cycling of bias voltages for a BaTiO_3_ layer with a thickness of δp = 3 µm. (**d**) The temporal evolution of the membrane occurs as the bias voltages are modulated at a frequency of 0.2 kHz, with turn-on values of Vg1 = Vg2 = 1 V. (**e**) Short-circuit pyroelectric current density. (Reproduced from [170], with permission).

Production of electricity from heat emission released by elevated temperature emitters was anticipated to significantly contribute to the utilization of waste heat [168]. The energy flow transferred between the primary source and the cell in these devices is fundamentally constrained by the Stefan–Boltzmann law, which governs the thermal flux between two blackbodies, hence establishing a relatively modest maximum for the power that is produced. Fang et al. [169] suggested an alternative method for harvesting near-field thermal energy with a pyroelectric converter. When the distance between source and the cell is in the subwavelength scale, near-field energy can be transmitted to the cell through the tunneling of non-propagating photons, allowing the heat flux to significantly surpass the constraints established by the blackbody theory. This method necessitates an active layer composed of a pyroelectric material that experiences a temporal temperature variation due to periodic modification of the distance between the layer and two external thermostats (the hot source and the cold sink). A distance of 100 nm, an operating frequency of few Hz, and an electric power of 6.5 mW cm^−2^ were anticipated with a source and sink at temperatures T_1_ = 383 K and T_3_ = 283 K, respectively. Recently, Latella and Ben-Abdallah [170] proposed a solution for increasing the performance of such devices by tackling the layer distance issue by a static pyroelectric converter which utilizes graphene field-effect transistor (GEFT) technology (Figure 14). Similar findings are also reported by Song and coworkers [171] on the application of ferroelectric materials and their pyro- and piezoelectric effect for sensing temperature and pressure simultaneously.

Sultana et al. [164] have proved the feasibility of water vapor-driven pyroelectric generators (PyG). The output performance of the PyG can be modulated by altering the effective working area of the device or by varying the frequency and amplitude of the temperature fluctuations. An open circuit voltage of 1.5 V, a short circuit current of 1.5 µA, and a power density of 0.034 µW/cm^2^ were recorded during a temperature variation from 310 K to 340 K. The PyG can function as a self-powered temperature sensor owing to its rapid response time of 121 ms. The electrical energy supplied by PyG from the energy contained in daily used hot water is stored in capacitors using a bridge rectifier circuit.

Miniaturization and multi-effects such as triboelectricity, piezoelectricity, and pyroelectricity allow the simultaneous use of self-powered wearable sensors for distinguishing external triggers (multipurpose sensing). Ma and coworkers [172] have achieved multimodal sensing of pressure and temperature by using a straightforward technique that couples the above-mentioned effects, by differentiating the voltage signal into two distinct voltage peaks with varying response times. The self-powered tactile sensor exhibits exceptional sensitivity of 0.092 V/KPa and 0.11 V/°C, respectively, exhibiting at the same time antibacterial properties and significant flexibility, enabling tactile monitoring on curved surfaces. Sun and coworkers [173] reported the use of a transparent tribo-piezo-pyroelectric hybrid energy generator as biomechanical energy harvester and physio-monitor.

Zhao et al. [174] introduced a versatile pyroelectric device that serves both as an efficient energy harvester for capturing waste heat from chemical exothermic processes and as a self-powered temperature monitor that reflects the chemical process in real time. The suggested device, consisting of a CNT/PVDF/CNT sandwich, can transform substantial and random heat generated from numerous chemical processes into electrical energy.

#### 4.6.2. Self-Powered IR and Pyroelectric Sensors

Roy and coworkers have developed a poly(vinylidene fluoride) (PVDF)-based self-powered, flexible hybrid nanogenerator (NG) device that integrates piezoelectric and pyroelectric properties. It may be affixed to various sites on human skin to detect both static and dynamic pressure variations, as well as to monitor temperature fluctuations during respiration. Doping with graphene oxide (GO) enhances the pyroelectric energy harvesting and sensing capabilities of the device over repeated temperature fluctuations. The PVDF/GO-based nanogenerator exhibits a maximum pyroelectric output power density of around 1.2 nW/m^2^ and is capable of detecting temperature variations during respiration, rendering it a suitable candidate for a pyroelectric breathing sensor. The manufacturing of the PVDF-GO self-powered multifunctional pressure and pyroelectric breathing sensor can be scaled up to produce tiny, high-performance electronic skins for health monitoring, motion detection, and portable electronics applications [175].

Wang et al. [176] proposed an innovative approach for active NIR sensing, which could have significant potential in biological imaging, optoelectronic communications, and optothermal detection. Near-infrared (NIR) photothermal-triggered pyroelectric nanogenerators utilizing pn-junctions are exhibited in a p-Si/n-ZnO nanowire (NW) heterostructure for self-powered NIR photosensing. The pyroelectric-polarization potential (pyro-potential) generated in wurtzite ZnO nanowires interacts with the intrinsic electric field of the pn-junction. Upon activating or deactivating the NIR illumination, an external current flow is generated by the time-varying internal electric field of the pn-heterostructure, facilitating a bias-free operation of the photodetectors (PDs). The NIR photodetector demonstrates a substantial on/off photocurrent ratio of up to 10^7^, together with a rapid photoresponse characterized by a rising time of 15 μs and a fall time of 21 μs.

#### 4.6.3. Self-Powered Wearable Sensors

The rapid advancement of wearable electronics, such as e-skins, necessitates self-powered sensing to harness various bio-energies from the human body and to monitor physiological data [173].

A practical strategy was proposed to simultaneously scavenge various types of energy from the environment through a hybridized energy harvester that incorporates two types of conversion cells for concurrent harvesting of solar and mechanical energies, thereby enabling effective and complementary utilization of energy resources. Triboelectric nanogenerators operate on the principles of contact electrification and electrostatic induction, and they have been extensively researched for the purpose of harvesting various forms of mechanical energy from the environment [177].

Considering all forms of energy harvesting technologies, transformation of thermal energy from heating waste is one of the most prevalent and readily accessible energy sources in our living environment and industrial operations. Pyroelectric nanogenerators (PyNGs) are developing as a potent instrument for harnessing lost heat. Xue et al. [178] have constructed a wearable PyNG utilizing PVDF thin film incorporated within a N95 respirator to harness energy from human respiration. Besides the impact of energy harvesting from human activities, electrically generated signals can also be used to check if the individuum exhibits a respiratory condition and act at the same time as sensors to measure the surrounding temperature. The devices are intelligent, intriguing, and hold significant potential for wearable energy harvesting, human health monitoring, and ambient temperature sensors. You and coworkers [179] demonstrated a lightweight and flexible self-powered hybrid nanogenerator utilizing the piezoelectric and pyroelectric effects of an electrospun non-woven poly(vinylidene fluoride) (PVDF) nanofiber membrane, which may function as an active layer without the need for post-poling treatment. The flexibility of the nanogenerator was improved by employing an electrospun thermoplastic polyurethane nonwoven fabric as a substrate, alongside a conductive PEDOT:PSS-PVP nonwoven fabric and a carbon nanotube layer as electrodes.

Compared to single crystals and bulk polycrystalline ceramic materials, inorganic thin films have lower thermal capacity and manufacturing costs. Thin films have exceptional structural features in the fabrication of flexible pyroelectric devices, rendering pyroelectric inorganic materials highly appropriate for self-powered flexible wearable electronics and sensor systems [12].

Nonetheless, the fabrication methods of these NGs are complex and laborious, posing hurdles to their bulk manufacture and practical use [179].

#### 4.6.4. UV/IR Detection and Imaging

Photodetectors possess extensive uses, including telecommunications, sensing, chemical analysis, thermal imaging, and biological imaging. Low-dimensional narrow-band-gap III–V semiconductors possess significant potential in high-performance electronics, photonics, and quantum devices. Nonetheless, high-performance nanoscale infrared photodetectors utilizing isolated two-dimensional (2D) III–V composite semiconductors remain scarce.

Narrow-band-gap III–V materials, including InAs, InSb, and InAsSb, have superior light detection capabilities across a spectrum ranging from near- and short-wavelength infrared (NIR and SWIR) to mid-wavelength infrared (MWIR) and beyond, in contrast to Si- and Ge-based photodetectors [180]. Due to the Fermi level of InAs being anchored toward the lower edge of its conduction band, InAs readily establishes Ohmic contact with many metals. InAs nanowires (NWs) have been utilized to construct many intriguing devices, including photodetectors, single-electron transistors, and ballistic transistors [181].

Wang et al. [182] have presented an innovative photodetector exhibiting exceptional responsivity (~1231 A/W), external quantum efficiency (EQE) of 2.2 × 10^5^%, and detectivity of 5.46 × 10^10^ Jones for 700 nm light, working at a low voltage of approximately 0.1 V, utilizing InAs nanosheets. The photodetector exhibits a high optoelectronic response across the ultraviolet-infrared spectrum (325–2100 nm) at ambient temperature.

Saleem and colleagues assert that colloidal quantum dots (CQDs) are highly promising nanomaterials for optoelectronics owing to their adjustable bandgap and quantum confinement effect. All-inorganic CsPbX_3_ (X = Br, Cl, and I) perovskite nanocrystals (NCs) have garnered significant interest due to their potential applications in solar systems. The workgroup developed all-solution-processed UV-IR broadband trilayer photodetectors based on ITO/ZnO/PbS/CsPbBr_3_/Au and ITO/ZnO/CsPbBr_3_/PbS/Au exhibiting high performance and analyzed the function of the CsPbBr_3_ quantum dots layer as the carrier-extracting layer in the trilayer devices. They claim that the all-solution-processed heterojunction design technique facilitates the development of high-performance broadband photodetectors as at present; reports on UV-IR broadband photodetectors utilizing all-solution-processed colloidal quantum dots with aligned energy gaps are scarce.

An interesting application is in the cultural heritage field, where currently two main applications for multispectral imaging are used [183,184]. Historically, the first goal has been to achieve high color fidelity. Depending on the application and its requirements, the employed devices use passive or active detection schemes and several kinds of sensors and radiation sources (lasers, X-ray tubes, halogen, and ultraviolet (UV) lamps, etc.). However, most of the current developments are focused on processing and analyzing the UV, visible, and near-infrared (IR) range images. A scientific cooled digital charge coupled device (CCD) camera (DTA) was used as an imaging device by Pelagotti et al. [183]. Its image sensor is a KAF 6303 e (Kodak), UV-enhanced, high quantum efficiency to 1100 nm, front-side illuminated transparent gate true two-phase technology sensor of 3072 × 2048 pixels, 9 × 9 micron. The sensor has a full well capacity of 100 ke, dark current of 0.5 e-/pixelsec, quantum efficiency at 450, 550, 650 nm of, respectively, 40%, 55%, 64%, and fill factor at 100%.

**Figure 15:**
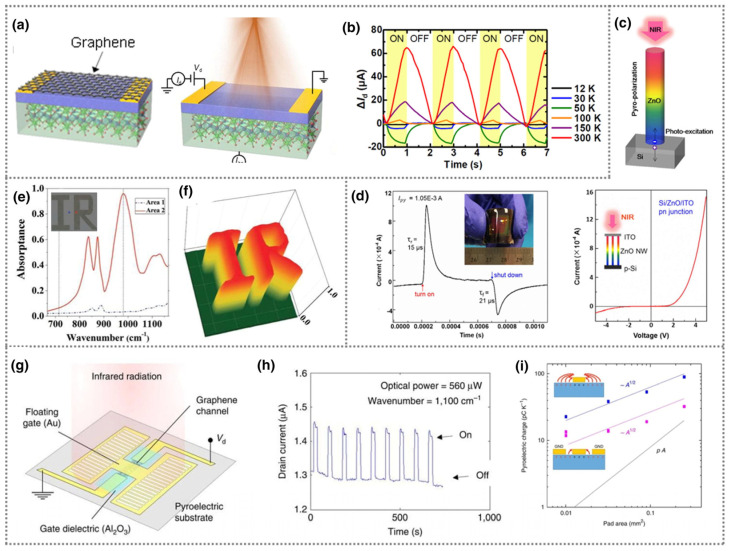
Devices utilizing pyroelectric materials for the detection and imaging of ultraviolet and infrared light. (**a**) Fabrication of LWIR graphene photodetectors utilizing LiNbO3 substrates featuring a graphene channel. (**b**) Variations in DId over time at different temperatures for Vd = 1 V. (**c**) NIR photodetectors exhibit specific structural and photoresponse characteristics, alongside instantaneous pyro-polarization and photo-excitation phenomena. (**d**) I-t curve under short circuit conditions and I-V characteristics for a single cycle of the pn-junction, along with optical images of the p-Si/n-ZnO NWs heterostructure-based device. (**e**) The experimental results indicate light absorptance and the specific area occupied by the metasurface-based absorber. Insight into a microscopic photograph of the metasurfaces. (**f**) Empirical absorption at 10.2 m in three-dimensional images. (**g**) Schematic representation of a single graphene pyroelectric bolometer device. (**h**) The drain current response is measured at a 10 mV drain voltage following multiple manually triggered ON/OFF cycles. (**i**) Measurements of the integrated pyroelectric charge per Kelvin for screened (magenta) and unscreened (blue) conditions. (Reproduced from [12], with permission).

In Figure 15, pyroelectric materials-based devices for the detection and imaging of UV and IR light are presented.

## 5. Summary and Outlook

The prospective applications of infrared detector systems necessitate enhanced pixel sensitivity, higher in pixel density; costs minimization of infrared imaging array systems through more efficient, or better, no cooling sensor technology and the integration of detectors with signal processing functions (featuring significantly increased on-chip signal processing); and advancements in the functionality of infrared imaging arrays via the development of multispectral sensors.

Numerous significant hurdles exist for prospective civilian and military infrared detector uses. In numerous devices, including night-vision goggles, the infrared image is perceived by the human eye, which can detect resolution enhancements only up to one megapixel, comparable to the resolution of high-definition television.

Array sizes will persist in expanding as expansion of array size is already technically achievable. Nonetheless, the commercial factors that necessitated larger arrays are not as robust now that the megapixel threshold has been surpassed.

QDIP-based infrared photodetectors are expected to offer benefits such as reduced dark current, elevated operating temperatures, normal incidence, and multicolor detection. Significant research has been conducted on QDIPs, and numerous advantages have been proven, yet this technology is hindered by low quantum efficiencies.

Currently, HgCdTe stands out as the most extensively utilized variable-gap semiconductor, holding a significant role in both the MWIR and LWIR spectral ranges. Theoretical predictions suggest that only type-II superlattice photodiodes and QDIPs are anticipated to rival HgCdTe photodiodes. Nonetheless, QDIPs are performing significantly lower than current HgCdTe detectors. Enhancing the uniformity of quantum dots is a crucial factor in elevating the absorption coefficient and optimizing overall performance as the weak performance of QDIPs is typically attributed to an inadequate band structure and a lack of uniformity in quantum dot size. The study of various strain-bed and capping material compositions surrounding quantum dots is crucial for enhancing quantum confinement in QDs. This can be achieved by reducing size and minimizing species migration during the growth of subsequent layers.

Advancements in nanofabrication will allow detectors to be produced cost-effectively, and the size and shape of the materials will allow for operation in longer wavelength bands and at elevated temperatures compared to their bulk equivalents. Bulk detectors, based on AlGaAs, HgCdTe, and SiGe, have been extensively commercialized owing to their ease of large-scale production, versatility in absorption wavelengths encompassing nearly all infrared bands, and elevated performance at cryogenic temperatures. Graphene and other two-dimensional materials have been extensively studied for their potential in higher working temperatures and reduced costs in infrared photodetector manufacture; yet, these novel materials exhibit a low absorption coefficient, heightened sensitivity to environmental conditions, and significant limitations in large-area fabrication. However, these low-dimensional materials possess the potential to exceed the performance of bulk semiconductors.

The Type-II strain layer superlattice is a highly advanced technology in infrared detectors. It possesses a variable bandgap similar to MCT, contingent upon the SL thickness and composition. Successful demonstrations of MWIR imaging arrays functioning at 120 K have been reported, achieving efficiencies nearing 40%.

A theoretical investigation of band-bending and carrier-transport effects near quantum dots will facilitate the precise design of barriers aimed at enhancing the performance of barrier devices. This includes the engineering of appropriate barrier designs, such as resonant tunneling barriers, confinement-enhancing barriers, and bound to quasi-bound barrier devices. Additionally, the escape probabilities of photoexcited electrons can be increased without compromising the absorption coefficients. DFT calculations can be helpful in understanding experimental results.

A vision for the future of infrared detector technology involves photodetectors paired with advanced electronics executing sophisticated algorithms where AI could be an important tool for adaptive systems. We might even observe arrays in which each pixel detects the entire infrared spectrum and also use in this respect bio-inspired solutions.

## Figures and Tables

**Table 1:** Properties of different organic and inorganic composite materials which includes Curie temperature T (°C), pyroelectric constant p (µC m^−2^ K^−1^), dielectric constant (ε_r_), and dielectric loss (tan δ). (Reproduced from [12] with permission).

Materials		Tc (°C)	p	ε_r_	tan δ	Materials		p	ε_r_	tan δ
PVDF	[15]	>80	27	12	0.015	8 vol% BaTiO_3_P(VDF/TrFE) 70/30	[16]	43	8	0.02
P(VDF/TrFE) 80/20	[17]	135	31	7	0.015	6 vol% LiTaO_3_-P(VDF/TrFE) 70/30	[16]	60	12	0.02
P(VDF/TrFE) 70/30	[18]		33	7.4	0.017	30 vol% (Bi_0.5_Na_0._5)0.94 Ba_0.06_ TiO_3_-P(VDF/TrFE) 70/30	[19]	47	21	
P(VDF/TrFE) 60/40	[20]		45	29		20 vol% Bi_0.5_Na_0.5_TiO_3_-P(VDF/TrFE) 75/25	[21]	50	~15	0.03
P(VDF/TrFE) 56/44	[22]		52	18	0.05	15 vol% K_0.5_Na_0.5_ NbO_3_-P(VDF/TrFE) 70/30	[21]	63	30	0.08
P(VDF/TrFE) 50/50	[23]	49	40	18		43 vol% TGS-P(VDF/TrFE)	[24]	102	12	
Polyvinyl fluoride (PVF)	[25]		18	5	0.015	20 vol% 0.88(Na_0.5_ Bi_0.5_)TiO_3_-0.084(K_0.5_ Bi_0.5_)TiO_3_-0.063BaTiO_3_-P(VDF/TrFE) 75/25	[21]	95	~19	0.47
Polyvinyl chloride (PVC)	[25]		1	5	0.01	50 wt% TGS-PVDF	[24]	~15	~7	
Polyacrylonitrile (PAN)	[25]		1	7.7	0.2	80 vol% TGS-PVDF	[24]	90	12	
26 vol% BaTiO_3_-HA	[26]		2	21	0.01	5 wt% DTGS-PVDF	[24]	38	16	
44 vol% BaTiO_3_-HA	[26]		21	38	0.02	20 vol% BaTiO_3_-PVC	[27]	32		
30 vol% KNbO_3_-HA PDMS	[28]		8			30 vol% BaTiO_3_-PVC	[27]	66		
5 wt% DM-BaTiO_3_ (nws)-PVDF	[29]		42	8	0.40	40 vol% BaTiO_3_-PVC	[27]	106		
7 wt% WO2.72 (nfs)-PVDF	[30]		40			30 vol% BaTiO_3_-rubber	[31]	60	17	

## Data Availability

Data sharing is not applicable to this article as no new data were created or analyzed.
